# Supplementary material for: Hybrid evolutionary machine learning model for advanced intrusion detection architecture for cyber threat identification
Source: PLoS One. 2024 Sep 12;19(9):e0308206. doi: 10.1371/journal.pone.0308206 (PMC11392230; doi:10.1371/journal.pone.0308206)
Supplement: Attachment — Submitted filename: Response Sheet for Plos.docx [file pone.0308206.s001.docx]

**TITLE: An Evolutionary Machine Learning Algorithm for Robust Intrusion Detection System for Identification of Cyber Attacks**

Authors : Ankita Sharma, Shalli Rani, Maha Driss

[ankita.sharma@chitkara.edu.in](mailto:ankita.sharma@chitkara.edu.in), shalli.rani@chitkara .edu.in, mdriss@psu.edu.sa

Dear Editors and Reviewers:

We are thankful to you for spending your valuable time for making a review and for constructing the comments on our manuscript. These comments are valuable and very helpful for revising and improving our paper. We have studied comments carefully and have made correction as marked in the revised manuscript. We have tried our best to address the mentioned comments to revise our manuscript in the hope that these revisions will meet your requirement. The following changes have been made in the manuscript as per the received comments.

*The changes made in the manuscript as per received comments have been highlighted in blue color.

Comment 1: In the ABSTRACT: The stated motivation is observed to be vague; the rationale behind the use of TWO datasets (BoT-IoT & UNSW NB 15) is NOT supported with scientific justification; what information does the paper intends to present through the choice of the performance metrics are NOT clear (and not stated); and generally, tautology pervades the entire presentation.

Response 1:

"In response to the rapidly evolving threat landscape in network security, this paper proposes an Evolutionary Machine Learning Algorithm designed for robust intrusion detection. We specifically address challenges such as adaptability to new threats and scalability across diverse network environments. Our approach is validated using two distinct datasets: BoT-IoT, reflecting a range of IoT-specific attacks, and UNSW-NB15, offering a broader context of network intrusion scenarios. This selection facilitates a comprehensive evaluation of the algorithm's effectiveness across varying attack vectors. Performance metrics including accuracy, recall, and false positive rates are meticulously chosen to demonstrate the algorithm's capability to accurately identify and adapt to both known and novel threats, thereby substantiating the algorithm's potential as a scalable and adaptable security solution. This study aims to advance the development of intrusion detection systems that are not only reactive but also preemptively adaptive to emerging cyber threats."

The following abstract has been updated by adding the new information as per comment and now the abstract is updated by keeping in mind all those points given by you.

Comment 2: In the INTRODUCTION: No definite research question is defined; again, throughout the paper, machine learning MODELS are erroneously being introduced as DOMAINS/systems/applications, whereas machine learning MODELS are PROGRAMS that are applicable to various DOMAINS (e.g., as in LINE 20, i.e., “...domains, including machine learning models ...”); also, the paper could not differentiate between genetic algorithm (GA) and genetic programming (GP); again, how could “... genetic algorithms inadvertently magnify the impact of adverse disturbances ...” (as in LINE 26); “... cybersecurity protocols advance in parallel with the machine learning algorithms they aim to safeguard ...” (as in LINE 38) is an example of the many UNFAMILIAR assertions; if “...Network Intrusion Detection Systems (NIDS) have been consistently improved to adapt to the dynamic nature of security threats ...” (as in LINE 44), what purpose does this study serves?; how the security concerns of smart city SPECIFICALLY relate to this research work is NOT clear; on the contributions, how F1 score harmonizes RECALL and ACCURACY is NOT clear, how FPR functionsas PENALTY PARAMETER is NOT clear, how the mean fitness between Bot-IoT and UNSW NB15 is evaluated is NOT clear and NOT reported, and the claimed speed is (as it is) SPECULATIVE; the current study has no SPECIFIC reference point to any SPECIFIC existing solution.

Response 2: As per the comment same has been added and highlighted. “This study investigates the question: How can evolutionary machine learning algorithms enhance the detection accuracy and adaptability of intrusion detection systems against evolving cyber threats”.

In line 20 phrase “A lot of domains” has been removed.

Line 26 has been updated by adding and highlighting “Genetic algorithms rely heavily on selection mechanisms that can, under certain conditions, lead to reduced genetic diversity within the population. This decrease in diversity might cause the algorithm to overfit to anomalies or disturbances in the data, potentially magnifying their impact and leading to less robust solutions.”

Line 38 cybersecurity protocols advance in parallel with the machine learning algorithms they aim to safeguard “the network from different cyber attacks” added and highlighted.

In Line 44 just a small intro to NIDS has been mentioned but no need of that, that is why same has been removed.

Actually the proposed methodology is designed for different applications such as smart cities, smart industries and etc, the same has been rephrased and highlighted.

F1 score harmonizes RECALL and ACCURACY where an imbalance between these metrics might lead to misleading interpretations of a model's accuracy has been added and highlighted.

High FPR indicates many benign activities are incorrectly classified as threats, which can lead to unnecessary alerts and operational inefficiencies. This disrupts user trust and increases the operational burden due to the need to investigate each false alarm, thereby acting as a penalty on the system’s usability and resource management has been added and highlighted.

Mean fitness for both BoT-IoT and UNSW NB 15 has been explained in algorithm as well as under section 4.4 as in this study is defined as the average performance score across multiple key metrics, including accuracy, precision, recall, and F1 score, calculated separately for each dataset. Same has been illustrated by Figure 3 and 4. Same has been added and highlighted.

Comment 3: In the LITERATURE REVIEW: No adequate focus on the SPECIFIC strengths, weaknesses, limitations, and/or challenges of EXISTING SOLUTIONS for Evolutionary Intrusion Detection System (to justify the need for the current study); instead, the SUPERFICIAL reviews (in one single paragraph) are focused on evolutionary approaches to feature selection, whose performances are mostly applauded; inadvertently justifying the dispensability of the current study. The preliminary understanding of evolutionary computing/programming/algorithms is expected to be strategically tucked into the INTRODUCTION section; or logically included in the REVIEWS.

Response 3: This Table numbered as 2 has been added in the related work and highlighted.

Also, preliminaries have been added under Introduction named as section 1.3 Preliminaries and highlighted.

| **Reference** | **Approach** | **Strength** | **Weakness** | **Limitation** | **Challenges** |
| --- | --- | --- | --- | --- | --- |
| [25] | Hybrid SVM-GA Model | High detection rate (0.973) and low false alarms (0.017) with reduced feature set. | May not perform as well with non-KDD'99 datasets. | Relies on optimal selection of features, which may not generalize across different attack vectors. | Ensuring robustness across diverse network environments and attack types. |
| [28] | MLP with PCA and GA | Reduces features to primary space, enhancing classifier efficiency. | PCA-selected features may lack detection capabilities without GA optimization. | Dependent on the initial PCA reduction quality. | Balancing dimensionality reduction and maintaining detection performance. |
| [31] | ANN-based IDS Model | Consistent detection performance without additional agents. | Long training times, which may impede timely deployment. | Performance capped without adding more agents; training data dependent. | Speeding up training processes while ensuring detection accuracy. |
| [48] | DE, PSO, GA Feature Selection | High precision in classification with DE (99.75% accuracy); effective feature reduction. | Training time variability; DE takes 1.62 seconds, which may be slow for real-time applications. | Optimal attributes selection may vary significantly with dataset changes. | Achieving fast and accurate feature selection in dynamic real-time scenarios. |
| [38] | Genetic Algorithm for DNN Architecture | Superior performance with fewer computational resources; optimizes DNN structure efficiently. | Might not adapt well to newer or unseen attack patterns. | Dependency on the genetic algorithm for optimal architecture can be resource-intensive. | Balancing computational efficiency with the adaptability of the DNN architecture. |

Comment 4: In the METHODOLOGY: No scientific approach is presented for hybridizing BoT-IoT and UNSW-NB15 datasets, and the properties of the hybrid dataset are NOT presented; most procedures are NOT assertive, as major steps sounds theoretically optional (e.g., on GA & DT: LINEs 307/309 & EQUATIONs 11/12 respectively, raises concerns/doubts on the paper’s experimental claims, because which condition actually applies to the current study is scholarly expected to be definitive for a properly concluded work); the functional property that the FITNESS function is exactly evaluating is NOT clear; most EQUATIONS are NOT expressively described; how the DT integrates into SVM (or vice-versa) is NOT presented; in context (as it is in the content of the reports), the methodology still remain PROPOSED; making replication/reproducibility evidently NOT feasible.

Response 4: For the hybridization and dataset details Reference 39 has been cited and highlighted.

[39] Rani, S., Bashir, A.K., Krichen, M. and Alshammari, A., 2023. A low-rank learning based multi-label security solution for industry 5.0 consumers using machine learning classifiers. *IEEE Transactions on Consumer Electronics*.

Same has been added and highlighted “The Hybrid DT-SVM Model-GAs, represents a sophisticated approach in machine learning that leverages the complementary strengths of DT, SVM, and the optimization power of GAs. This model integrates the intuitive decision-making process of DTs with the robust classification capabilities of SVMs to handle both linear and complex non-linear patterns effectively. The integration begins with GAs selecting the optimal subset of features from the dataset. GAs operate through a process of evolution-inspired operations including selection, crossover, and mutation, continually refining the feature set by evaluating its performance using a fitness function typically based on the accuracy or the F1 score generated by the hybrid DT-SVM model.

The DT component of the hybrid model excels in managing categorical data and explicit decision-making by recursively partitioning the data, thus making the model interpretable and efficient in handling varied data types. On the other hand, the SVM component is utilized for its capability to efficiently classify high-dimensional spaces and its effectiveness in scenarios where the separation margin between classes is vital. The synergy between DT and SVM in this hybrid model is crucial, as it allows for the leveraging of DT's handling of specific feature interactions while SVM provides robustness against overfitting, especially in complex classification landscapes.

This hybrid model is trained on the selected features, ensuring that each algorithm plays to its strengths, thereby enhancing the overall predictive performance. The GA's role is critical as it dynamically searches for the most effective combinations of features that maximize model accuracy, thus directly influencing the model’s capacity to generalize well on unseen data. The convergence of these techniques into a single framework presents a powerful tool for tackling complex predictive tasks in fields ranging from cybersecurity to medical diagnostics, where both precision and interpretability are paramount.”

For Equation 11 and 12 actually Ive described the DT in elaborated form using entropy and gini index, but not experimenting with the same. That is why it is better to be removed. I removed both the equations. Priorly it was added because description of DT is somewhere incomplete without Gini Index and Entropy. But its ok, even after removing it as per your valuable comment.Top of Form

**Comment 5:** In the RESULTS/DISCUSSION: Though their findings (results) are expected in this section, the scientific basis for the formulation of the temporal and spatial complexity METRICS are mandatory method issues; more methodical presentations are found in this section (such as Sections 5.3/5.4, Figure 2, Tables 2/3); even section 5.2 analyses the method (NOT the findings), so NO scientific discussion is presented.

**Response 5:** Consequently, the overall temporal complexity of the suggested method is O(g(p_{size} × c^2_{len})). The space complexity is O(c^2_{len}). The explanation is already there in Results. I ve already explained the analysis results and discussion in the paragraph form and also in the tabular form of which values has been taken for the experiment.

**Comment 6:** In the CONCLUSION: Table 4 is expected to feature under DISCUSSION, while Figures 4/5/6 are items under RESULTS; claims are NOT supported with data, with NULL references to NONEXISTENT values(e.g., “... features are chosen using GA using the settings that are provided below ...”); which ITEM (in particular) is advised/considered (as in LINEs 484 and 490); works under comparison are DISSIMILAR (as NONE of them employed GA, some of them utilized unrelated classifiers, and most of them adopted single classifier/dataset); the claim that the current intrusion predictions are better than the predictions of other previous existing solutions should be based on SIMILAR circumstances/conditions.

**Response 6:** Following changes has been made “Table 4 is put under DISCUSSION, named as Table 5, because I added Table 2 in Related work and Figures 4/5/6 are items under RESULTS already”; The following lines has been fixed and irrelevant has been removed.

**Comment 7:** In GENERAL: Although the language (grammar) is good, inconsistencies, incoherencies, contradictions and ambiguities are OBSERVED to have pervaded the entire manuscript (e.g., ambiguities in Sections 4.2/4.3); Figure 6 is referenced as Figure 7 (in LINE 467) and as Figure 9 (in LINE 469); Figure 5 is referenced as Figure 6 (in LINE 467): Figure 7 (whose dataset is NOT specified, and which is PRESENTED in the Reference section) is referenced as Figure 8 in LINE 468; Figures 3 & 4 are referenced as Figures 4 & 5respectively; Figure 2 is referenced as Figure 3 (in LINE 454); Bot-IoT dataset has more than 29 features (as the choice of that 29 is NOT justified); the Figure 2 (which is referenced in LINE 391) does NOT exist; equations (15) & (13) are referenced as references [15] & [13] in LINEs 377 & 349 respectively; the equation4) (which is referenced in LINE 327) does NOT exist; NULL references are observed (e.g., such as “below” in LINE 483); most EQUATIONS are NEVER referenced; most cited works are NOT relevant to the context of discussion (e.g., such as [2] in LINEs 12 vs 517, [37]/[38] in LINEs 243 vs 612/614, and [3] in LINEs 14 vs 519); references [33], [48], [49], [34] are disorderly cited; references [50], [39] – [44] are disorderly cited; reference [59] cited, but NOT listed; Quinlan et al. in 1987 and Gelfand et al. in 1991 are cited, but NOT listed; Vapnik et al. cited (NOT dated), and NOT listed.

**Response 7:** All the relevant changes have been made. All the figure numbers are updated, references are in sequence and updated,added reference numbers at the last of the equations, Two equations of Gini Index and Entropy has been removed. Also no figure is cited in the REFERENCE, as per the template some of the figures are showing that way.
